# Reduction of IL‐6 gene expression in human adipose tissue after sleeve gastrectomy surgery

**DOI:** 10.1002/osp4.396

**Published:** 2020-01-15

**Authors:** Isabel Casimiro, Erin C. Hanlon, Jeremy White, Avelino De Leon, Ruby Ross, Katiannah Moise, Matthew Piron, Matthew J. Brady

**Affiliations:** ^1^ Department of Medicine, Section of Endocrinology, Diabetes & Metabolism University of Chicago Chicago Illinois; ^2^ Committee on Molecular Metabolism & Nutrition University of Chicago Chicago Illinois

**Keywords:** bariatric surgery, interleukin‐6, macrophages, obesity

## Abstract

**Objective:**

There is increasing evidence that immune cell interactions in adipose tissue contribute to the development of metabolic dysfunction. Pro‐inflammatory cytokines have been shown to mediate insulin resistance, and the presence of macrophages is a salient feature in the development of obesity. The present study aimed to evaluate adipocyte size and macrophage activation in women before and 3 months after laparoscopic vertical sleeve gastrectomy (VSG).

**Methods:**

Subcutaneous abdominal adipose tissue biopsies were obtained from women scheduled to undergo VSG. Histological evaluation of adipocytes and macrophages was performed as well as cytokine expression quantification before and after VSG‐induced weight loss.

**Results:**

Weight loss following VSG resulted in a reduction in adipocyte size as well as a decrease in interleukin (IL)‐6 cytokine mRNA expression in subcutaneous adipose tissue. There was no change in the presence of crownlike structures after weight loss.

**Conclusions:**

Early weight loss after VSG is associated with a reduction in adipocyte size and a decline in IL‐6 gene expression in local adipose tissue. Macrophage infiltration and crownlike density structures persist in adipose tissue from tissues impacted by excess body weight 3 months after VSG‐induced weight loss.

## 1 INTRODUCTION

Activated immune cells play a pathogenic role in the development of obesity and contribute to the development of systemic insulin resistance.^1^, ^2^ Multiple studies have implicated the accumulation of adipose tissue macrophages (ATMs) and pro‐inflammatory cytokines in the inflammatory response in obesity.^3^, ^4^ Increased lipid storage in the setting of obesity leads to adipocyte hypertrophy, which is associated with metabolic dysfunction including the development of insulin resistance.^5^ Cytokines originating from inflamed hypertrophied adipose tissue lead to monocyte infiltration and subsequent polarization of macrophages towards an inflammatory phenotype characterized by production of pro‐inflammatory cytokines such as interleukin (IL)‐6, TNF‐α, and IL‐1β.^6^, ^7^, ^8^, ^9^, ^10^ As adipocytes become hypertrophic in the context of obesity, they increase production of leptin, an adipokine involved in the regulation of feeding behaviour in the CNS in order to suppress food intake and limit adipocyte expansion.^11^ Circulating levels of leptin increase as adipose tissue expands and as such positively correlates with the degree of obesity.^12^ However, an eventual resistance to leptin develops, yet the pro‐inflammatory effects of leptin persist and perpetuate a state of chronic low‐grade inflammation.^3^, ^8^, ^13^ IL‐6 is a major inflammatory mediator that has been implicated in obesity in several studies.^14^ Aside from its important immune function, it is a pleotropic cytokine that is secreted by various cell types including skeletal muscle and adipocytes, which can mediate distinct biologic functions depending on where it is secreted.^15^ For instance, levels of IL‐6 can increase systemically after exercise. However, the majority of exercise induced IL‐6 arises from skeletal muscle rather than adipose tissue.^16^ Indeed, it has been demonstrated that IL‐6 released from skeletal muscle has beneficial effects on insulin resistance because of an increase in lipolysis and fat oxidation and an increase in glucagon like peptide‐1 (GLP‐1).^17^ By contrast, IL‐6 derived from adipose tissue in the context of obesity leads to hepatic insulin resistance via activation of suppressor of cytokine signalling‐3 (SOCS3).^18^, ^19^ Levels of IL‐6 acutely rise as part of an early response to inflammation or traumatic injuries, while chronic low‐grade inflammation involving increased circulating levels of IL‐6, and other cytokines such as TNF‐α and IL‐1β, are a known driver of metabolic disease.^20^

ATMs can comprise over half of the leukocytes present in adipose tissue.^3^, ^21^ One role of ATMs in tissues impacted by obesity is to participate in the clearance of dead adipocytes. However, ATM clusters are also seen during acute caloric restriction.^22^ Thus, they may also play an important role in lipid clearance during weight loss.^23^ Crownlike structures (CLSs) are formed by macrophages coalesced around dead hypertrophied adipocytes in inflamed adipose tissue, which are mainly seen in the context of obesity, and have been associated with increased insulin resistance.^24^, ^25^, ^26^ This suggests that weight loss should lead to a reduction in inflammatory mediators, macrophages, and CLS. Numerous studies have shown that bariatric surgery reduces insulin resistance, cardiovascular disease, and mortality independent of weight loss, effects thought to be related to changes in gut hormones and a reduction in inflammation seen after bariatric surgery.^27^ However, the finding that pro‐inflammatory mediators decrease after bariatric surgery‐induced weight loss has been inconsistent across multiple studies.^28^, ^29^, ^30^ A few studies have shown a reduction in subcutaneous adipose tissue gene expression of IL‐6 six months after bariatric surgery.^31^, ^32^ After bariatric surgery‐induced weight loss, systemic IL‐6 levels have been reported to be more variable: either reduced or unchanged.^33^, ^34^, ^35^ Differences in type of bariatric surgery, tissue source, patient age, and/or follow‐up duration may account for some of these inconsistencies. However, there is a limited amount of data regarding the early changes that occur in chronic low‐grade inflammation after a sleeve gastrectomy. Here, adipose tissue was evaluated from women with obesity before and early after vertical sleeve gastrectomy (VSG) to determine changes in parameters related to inflammation, such as inflammatory cytokine gene expression, systemic cytokines, ATM infiltration, and presence of CLS.

## 2 METHODS

### 2.1 Patient selection

This was a prospective study that included a total of 12 patients with obesity having a body mass index (BMI) above 35 kg m^−2^ who were previously scheduled to undergo laparoscopic VSG at the University of Chicago Center for the Surgical Treatment of Obesity, Chicago, IL (Table 1). Not all 12 patients returned for a post‐VSG subcutaneous fat biopsy or laboratory assessment. Because of limited tissue availability, the number of patients included in each experiment is denoted in the respective figure legend and includes pre‐VSG and post‐VSG assessments of the respective patients. Only women were recruited in the study in order to ensure proper powering, since the majority of patients who obtained a VSG at the University of Chicago are women. Patients were excluded from the study if they had pre‐existing diabetes, were on drugs known to alter insulin sensitivity, or had untreated obstructive sleep apnoea, which is also known to alter insulin sensitivity. Patients who were selected for VSG met with a multidisciplinary team for assessment that included surgeons, anaesthesiologists, bariatric physicians, and dieticians. Demographic information, vital signs, bioimpedance information, and blood samples were collected before and 3 months after VSG. Bioimpedance was assessed using the Quantum X BIA analyser validated in this population as previously described.^36^ Data included weight, height, and body fat mass values. Blood samples were collected from each patient in the morning in a fasted state and stored at −80°C until sample analysis. Patients were recruited between 2015 through 2018, were between 20 and 50 years of age, and provided written informed consent. The protocol was approved by the Institutional Review Board at the University of Chicago.

**Table 1:** Demographics and characteristics of the 12 patients who participated in the study

	Participants (n = 12)
N, race	9 non‐Hispanic black, 3 non‐Hispanic white
Age	37.4 ± 8.1

### 2.2 Subcutaneous fat biopsy

For the subcutaneous abdominal fat biopsy, an area of 12 to 15 cm in diameter was prepped with betadine solution. Lidocaine field block was performed using 20 cm^3^ of 1% lidocaine and anaesthetized for 5 to 10 minutes. An incision was made approximately 0.5 cm parallel to the waistline, and prefilled 60‐cm^3^ syringes containing 10‐cm^3^ saline solution were used to aspirate adipose tissue using up to six syringes with a 13‐gauge 3‐in. hypodermic needle, and approximately 8 to 12 mL of adipose tissue was obtained. Samples were removed from the syringes under sterile conditions and processed for further analysis as described below. Subcutaneous adipose tissue biopsies were performed on 10 paired patients 1 to 2 weeks before and 3 months after undergoing VSG.

### 2.3 Histological analysis of adipose tissue

Subcutaneous adipose tissue was fixed with formalin and embedded in paraffin for serial sectioning. Adipocyte morphology was evaluated by immunohistochemistry (IHC) and immunofluorescence (IF) techniques using subcutaneous adipose tissue sections from six patients before and after VSG as previously described.^37^ Stored 5‐μm sections were stained with antibodies against perilipin (PLIN2, 1:100, Abcam), as both PLPN1 and PLPN2 surround the adipocyte membrane.^38^ Appropriate secondary antibodies conjugated to a fluorophore (DyLight 594, Abcam) were used for IF. The sections were costained with Mac2 (1:200) macrophage primary antibodies (Cedarlane) for macrophage analysis and respective secondary antibodies conjugated to a fluorophore (Alexa Fluor 488, Abcam). Fluorescence images were acquired using an Olympus FV1000 confocal microscope. The cross‐sectional area was measured as an indicator of adipocyte size using CellProfiler software. The average adipocyte cross‐sectional area was calculated from each patient, and measurements were obtained from at least seven randomly selected fields from at least three slides per subject representing different sites of adipose. Adipocyte area measurements from each patient are presented as size distributions in Figure 2. Macrophage infiltration was assessed by CellProfiler software by counting Mac2‐positive signal in at least seven randomly chosen fields from three different slides representing different sites of adipose. Given that the number of adipocytes per field changed before and after VSG, macrophage signal was calculated as percent stain per field and then normalized to adipocyte number as previously described.^39^ For CLS count, given CLS density is less ubiquitous in human fat compared with that of mice, at least four slides were used in the analysis representing different adipose tissue sites for total CLS count. CLS density was compared in six paired patients before and after VSG by counting CLS numbers in the entire section. Four to six slides were used to represent different sites of adipose tissue. CLSs were defined as a Mac2‐positive circular signal coalesced around an adipocyte. A circular Mac2‐positive signal met criteria for CLS if the arc was at least 180° around an adipocyte. The numbers of CLS per section were recorded by an independent observer blind to the condition and presented per total area of adipose tissue measured by ImageJ and presented as CLS per square centimetre.

### 2.4 Gene expression

Adipose tissue was finely minced, and samples were allowed to recover for 12 hours in a 37°C 5% CO_2_ incubator in M199 media with 1nM of insulin and 40nM of dexamethasone as previously described for adipose tissue cell culture.^40^ mRNA was isolated from adipose tissue using the Omega Bio‐tek E.Z.N.A. Total RNA Kit II. The purity of RNA was confirmed by spectrophotometric analysis, and the quality assessed by Agilent 2100 Bioanalyzer. cDNA was generated by a reverse transcriptase reaction using qScript cDNA Supermix kit. Human primers were obtained for IL‐6, IL‐10, IL‐1β, and TNF‐α (RT^2^ qPCR Primer Assay, QIAGEN). Cytokine gene expression was assessed by real‐time quantitative polymerase chain reaction (PCR) using the Biorad CFX Connect Real Time System and SYBRGreen Master Mix (Agilent). PCRs were performed in triplicate in 96‐well plates, The data were analysed with the ddCt method as previously described,^41^ after normalization to an internal control, tyrosine 3‐monooxygenase/tryptophan 5‐monooxygenase activation protein, zeta (YWHAZ), a routinely used housekeeping gene for studies in adipose tissue.^42^ Levels of the YWHAZ transcript did not change after the intervention. The results were expressed as fold change for each target gene from baseline. Real‐time PCR analyses were performed using 10 paired patients before and after surgery.

### 2.5 Systemic cytokine quantification

Human cytokine quantification was performed using a Milliplex MAP kit for Human Cytokine/Chemokine magnetic bead panel, which measured five analytes in serum from nine paired patients before and 3 months after VSG. Six of the nine patients from this cohort also obtained a fat biopsy for mRNA analysis of adipose tissue. Serum samples were obtained in the morning after an overnight fast. Analytes included in the kit were IL‐6, IL‐10, IL‐β, and TNF‐α. All samples were acquired on a Luminex 200 instrument (Millipore) and were performed in duplicate in 96‐well plates. The serum concentration of leptin was determined by radioimmunoassay using the human leptin RIA kit (Millipore). The absorbance values were used to calculate the leptin concentration and were carried out in duplicate, and the average of the two values was used for data analysis.

### 2.6 Statistical analysis

Fat processing for immunohistochemical studies was performed in six paired patients before and after VSG. Because of limited sample availability, only 10 paired patients were used for mRNA gene expression studies and nine paired patients for the serum cytokine assays. Descriptive statistics were used to summarize demographic and clinical information. Linear regression was used to determine correlations between IL‐6 levels and body fat using Pearson correlation coefficients. Analyses utilized paired *t* tests for comparison of the change in each study endpoint as a result of VSG‐induced weight loss after 3 months. Data are presented in mean ± SD, and a value < .05 was considered statistically significant. Comparison and statistical tests were performed using GraphPad Prism (Version 8.2.1 for Windows 64‐bit).

## 3 RESULTS

**Figure 1:**
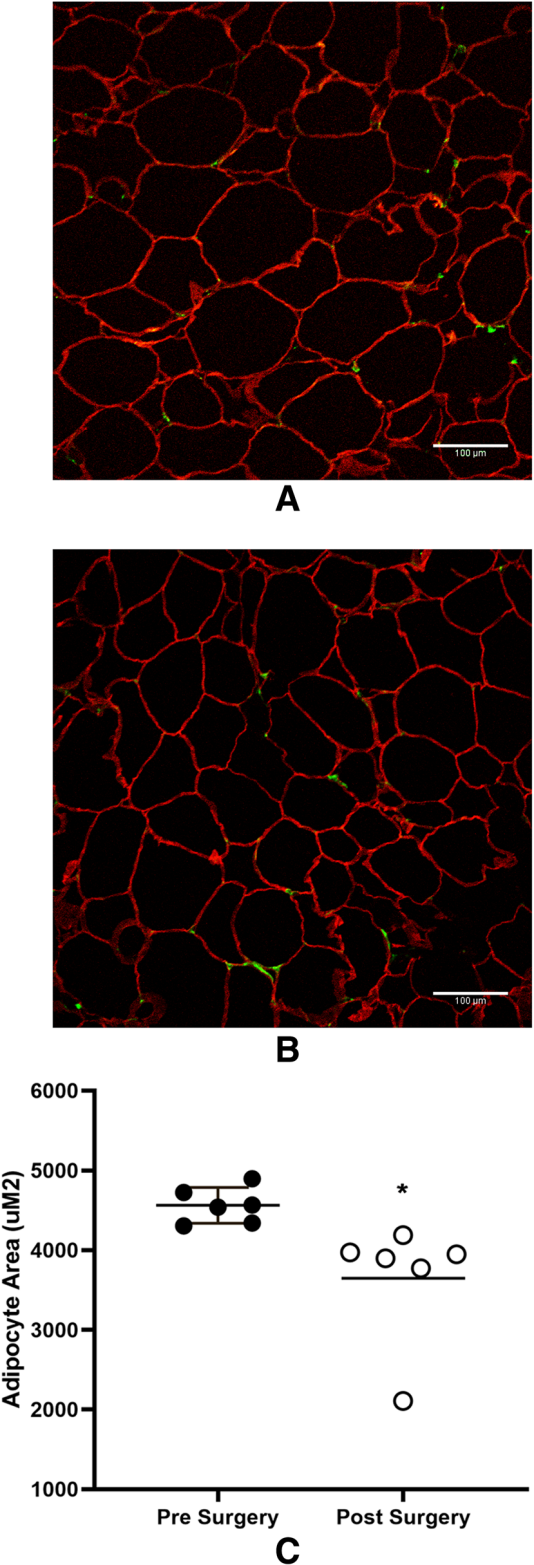
A, Adipocyte size. Immunofluorescence demonstrating adipocyte size from one patient, 1 week before vertical sleeve gastrectomy (VSG). B, Adipocytes 3 months after vertical sleeve gastrectomy surgery (red, antiperilipin surrounds the adipocyte; green, anti‐Mac2 Ab staining for macrophages; scale bar, 100 μm; images taken at 40× magnification). C, Mean adipocyte area from individual patients 1‐2 weeks before (black circles) and 3 months after (white circles) VSG (cell size quantified using CellProfiler), n = 6, *P < .05

**Figure 2:**
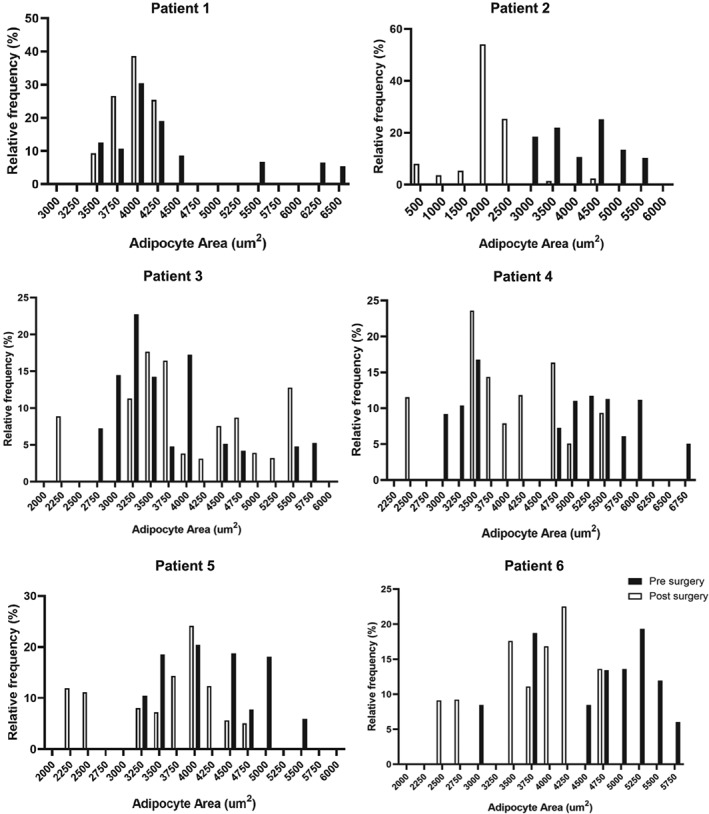
Adipocyte size of individual patients presented in size bins before and after vertical sleeve gastrectomy (VSG) induced weight loss (adipocyte area on the *x*‐axis is presented in bins of 250 or 500 μm^2^ as depicted in each figure; percent frequency in each size bin is presented on the *y*‐axis; black bars, presurgery; white bars, postsurgery)

The mean weight loss in women who underwent VSG was 21.51 ± 4.2 kg at 3 months (Table 2), which translated to a mean of 16.45 ± 2.94% of total body weight loss. Figure 1A demonstrates histological assessment of adipocyte hypertrophy by IF of a representative field of adipose tissue in one patient before VSG (Figure 1A) compared with 3 months after VSG‐induced weight loss (Figure 1B). Figure 1C shows the mean adipocyte area averaged for each patient before and after VSG‐induced weight loss at 3 months (n = 6). Figure 2 depicts the individual change in adipocyte area before and after VSG for each patient. There was a significant reduction in mean adipocyte area 3 months after VSG‐induced weight loss (−915.7 ± 636.5μM, *P* = .017; Figure 1C).

**Table 2:** Weight and BMI change from patients with obesity before (1‐2 wk) and after (3 mo) vertical sleeve gastrectomy

	Presurgery	Postsurgery	Change	P Value
Weight (kg)	131 ± 18.5	110.4 ± 17.4	−21.51 ± 4.2	<.0001
BMI	47.67 ± 6.6	40.05 ± 6.1	−7.62 ± 1.6	<.0001
Body fat (%)	51.91 ± 4.6	47.66 ± 4.2	−4.25 ± 1.7	<.05

*Note.* Values are presented as means ± standard deviation, n = 12.

Abbreviation: BMI, body mass index.

**Figure 3:**
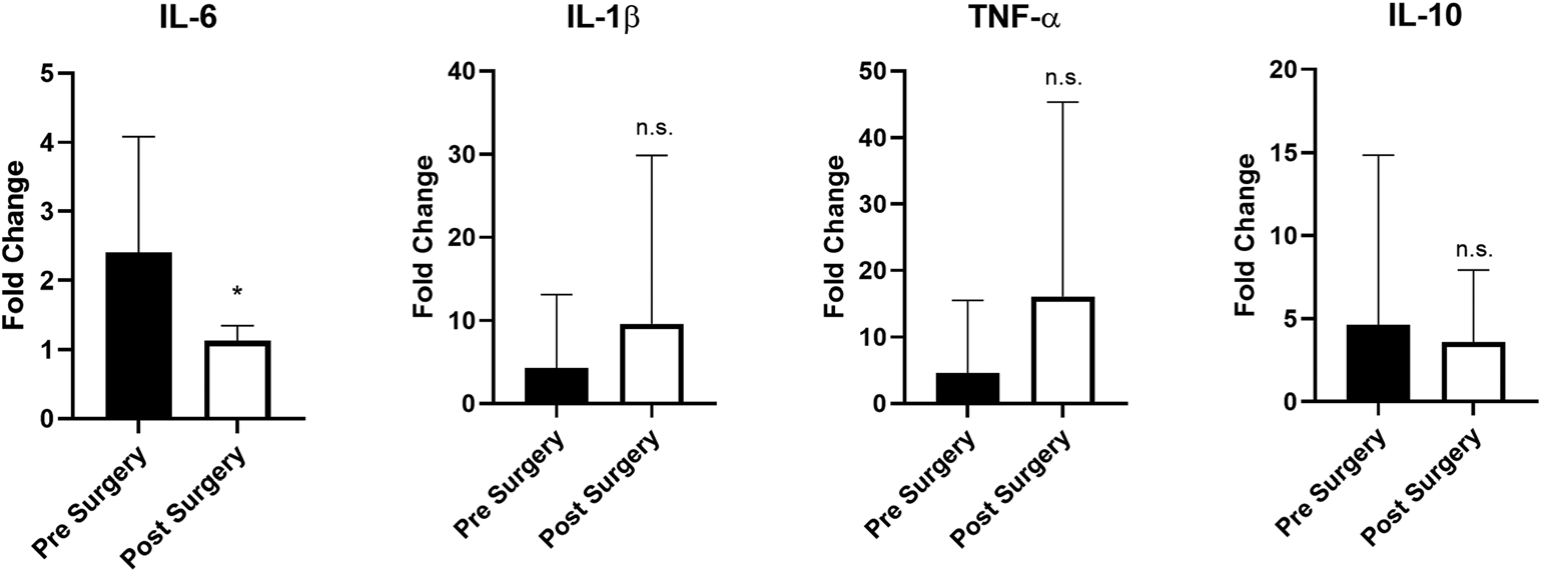
Adipose tissue cytokine gene expression. Expression of IL‐6, IL‐1β, TNF‐α, and IL‐10 in subcutaneous adipose tissue of subjects before (1‐2 weeks) and 3 months after bariatric surgery (*y*‐axis denotes relative gene expression expressed as fold change, n = 10, *P < .05; ns, not significant)

**Figure 4:**
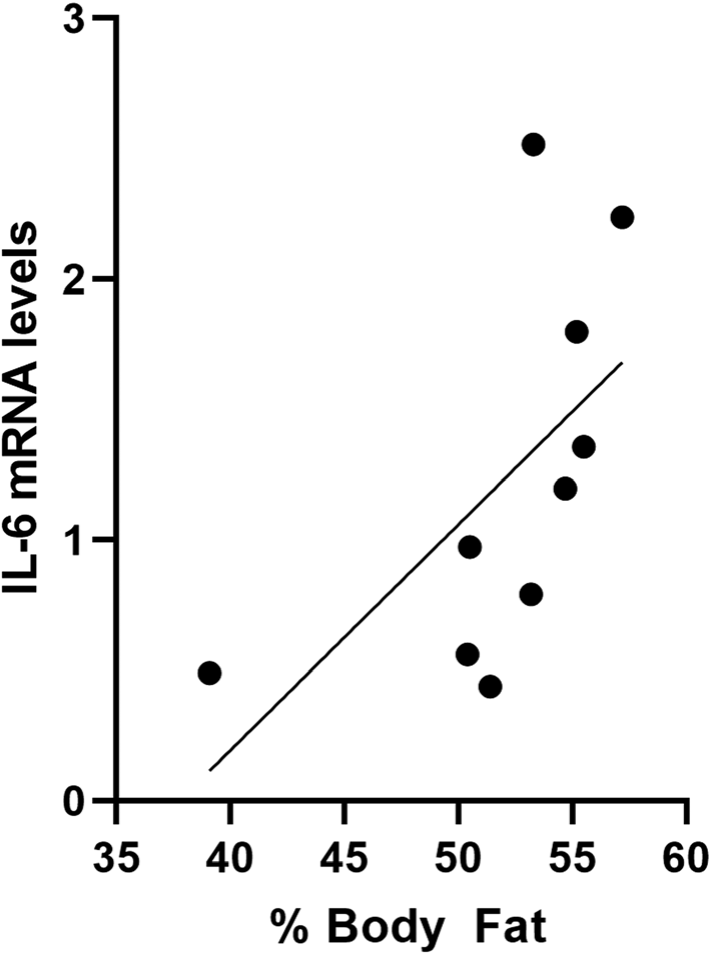
Correlation between levels of IL‐6 mRNA levels of female patients with obesity before vertical sleeve gastrectomy (VSG) and percent body fat (*P* = .069, *R* ^2^ = .354, 95% CI, −0.0549 to 0.891, n = 10)

Gene expression was analysed from mRNA using adipose tissue obtained from 10 patients who underwent subcutaneous fat biopsy before and 3 months after VSG. There was a statistically significant 2.1‐fold reduction (*P* = .02) in IL‐6 gene expression postsurgery, compared with presurgery levels. No significant changes were observed in IL‐1β, TNF‐α, or IL‐10 gene expression from adipose tissue (Figure 3). Given the reduction of IL‐6 expression in adipose tissue in the setting of weight loss, correlation analyses were performed between IL‐6 gene expression and fat mass. There was a positive correlation between mRNA levels of IL‐6 gene expression in adipose tissue and amount of body fat percentage from patients with obesity before surgery, which was close to reaching statistical significance (Figure 4, *P* = .06, *R* ^2^ = .354).

**Figure 5:**
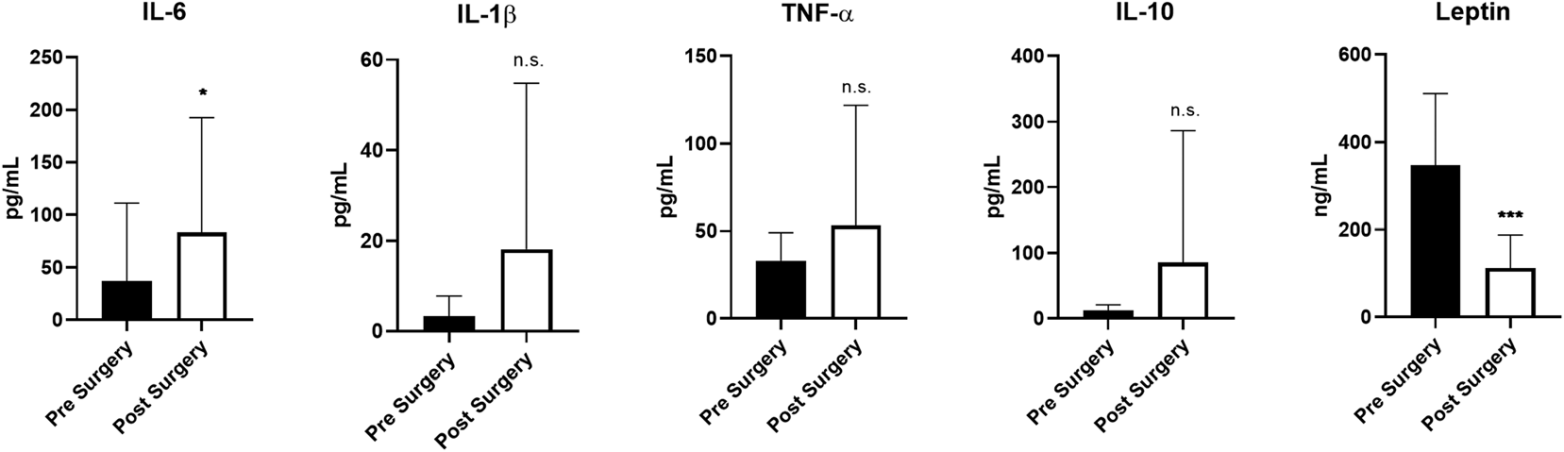
Systemic cytokine and adipokine levels from patients 1‐2 wk before vertical sleeve gastrectomy (VSG) and 3 mo after VSG‐induced weight loss (***P < .001, *P < .05; ns, not significant, n = 9)

Next, systemic cytokine and adipokine levels were measured in nine paired patients 1 to 2 weeks before and 3 months after VSG. There was an increase in IL‐6 serum levels, which reached statistical significance (36.90 ± 74.2 pg mL^−1^ pre‐VSG compared with 83.67 ± 109.1 pg mL^−1^ post‐VSG, *P* = .029, Figure 5). There was no change in systemic levels of IL‐1β, TNF‐α, or IL‐10 after VSG‐induced weight loss at 3 months. Many studies have shown significant reductions in leptin levels early after bariatric surgery‐induced weight loss.^43^, ^44^ Circulating leptin levels fell from 346.8 ± 165.1 ng mL^−1^ before VSG to 112.6 ± 75.47 ng mL^−1^, *P* = .0006. Our results confirm the finding that leptin levels decline early after VSG‐induced weight loss.

**Figure 6:**
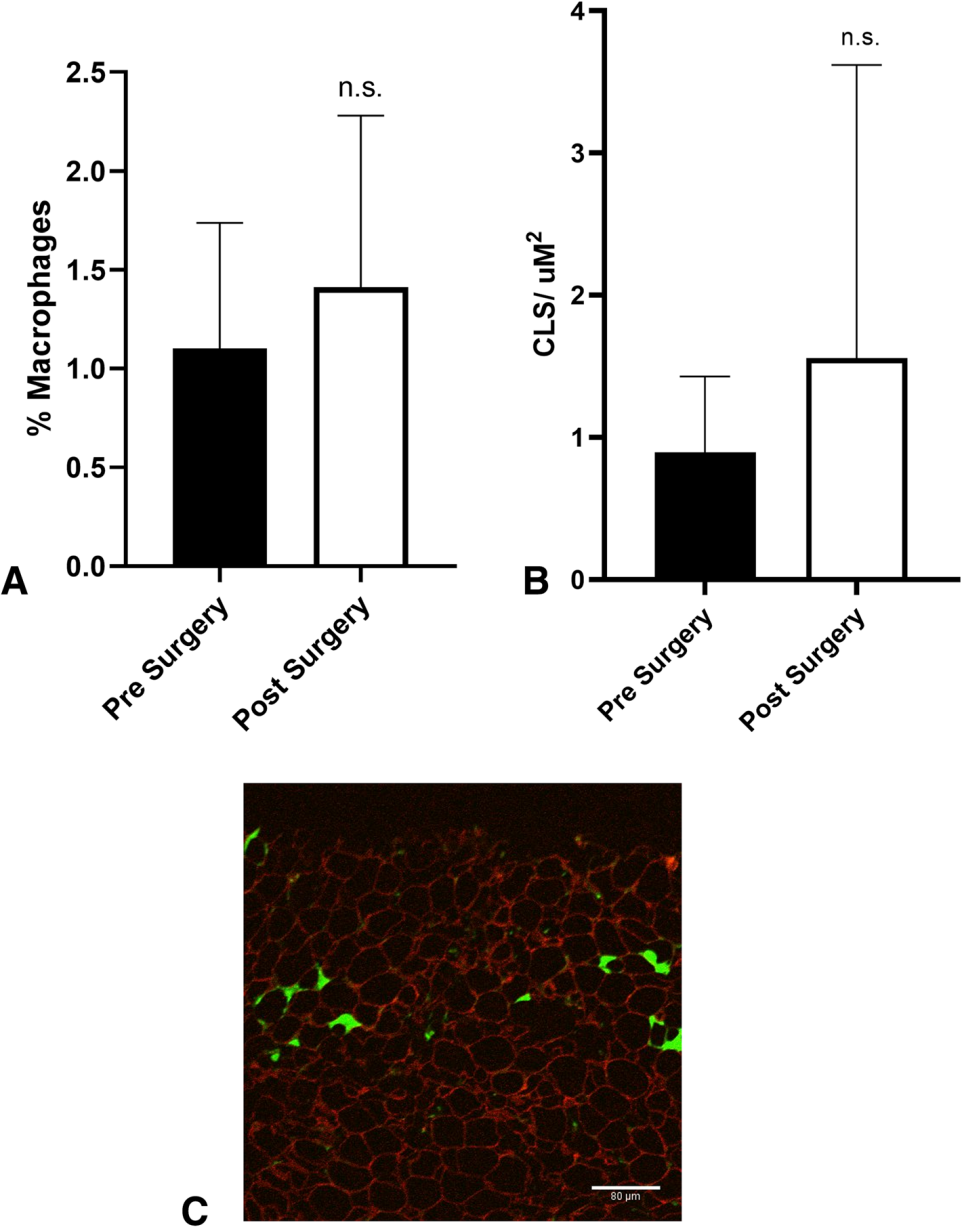
A, Macrophage infiltration in subcutaneous adipose tissue from patients 1‐2 weeks before and 3 months after vertical sleeve gastrectomy (VSG) (n = 6; ns, not significant). B, Crownlike structure (CLS) density from subcutaneous adipose tissue sections collected from patients 1‐2 wk before and 3 mo after VSG (n = 6; ns, not significant). C, Representative field from patient showing CLS density 3 months after VSG (red, anti‐perilipin surrounds the adipocyte; green, anti‐Mac2 Ab staining for macrophages; scale bar: 80 μm; image taken at 20× magnification)

Macrophage infiltration was assessed in subcutaneous adipose tissues using IF. Macrophage infiltration was observed before and after VSG‐induced weight loss. The percentage of macrophage infiltration was not significantly different when paired patients were grouped before or 3 months after VSG (Figure 6A). CLS density was evaluated from fat biopsy sections. When patients were grouped, there was no significant difference in number of CLS density observed before surgery compared with after VSG‐induced weight loss at 3 months (Figure 6B, representative field shown in Figure 6C).

## 4 DISCUSSION

The present study showed that VSG results in significant weight loss and reduction of body fat mass 3 months after bariatric surgery. Levels of the pro‐inflammatory cytokine IL‐6 decreased significantly at the mRNA level in subcutaneous abdominal adipose tissue from patients 3 months after VSG‐induced weight loss. This is in line with other reports that show that IL‐6 expression is associated with obesity. Furthermore, a positive correlation trend was observed between obese adipose tissue and mRNA IL‐6 levels, which may suggest that adipose tissue is the source of IL‐6 production in the state of obesity. However, levels of IL‐6 at the systemic level increased 3 months after VSG‐induced weight loss, showing that there is a distinct mechanism of IL‐6 regulation occurring at the local compared with systemic level, likely involving other tissues. While studies have shown a reduction in pro‐inflammatory cytokines including IL‐6 one year after sleeve gastrectomy^45^ or no change in IL‐6 serum levels 6 months after VSG or Roux‐en‐Y gastric bypass (RYGB),^46^, ^47^ to our knowledge, levels of systemic IL‐6 have not been shown to increase after VSG‐induced weight loss.

Reduction of IL‐6 in adipose tissue after VSG‐induced weight loss supports the finding that weight loss may be related to a reduction in local pro‐inflammatory cytokine production in adipose tissue. However, systemic levels of IL‐6 have been shown to be to be increased in subjects with obesity and to be predictive of the development of type 2 diabetes.^48^ Yet IL‐6 deficiency in mice results in obesity, hepatosteatosis, insulin resistance, and liver inflammation^49^, ^50^; and in humans, infusion of IL‐6 improves insulin sensitivity.^51^ The observation of increased systemic levels of IL‐6 after VSG‐induced weight loss suggests that IL‐6 may play a beneficial role in insulin sensitivity systemically early during weight loss.

The present study showed that there was no change in macrophage infiltration in adipose tissue after acute weight loss. While several studies have shown that macrophage activity has been associated with obesity and reduced systemic measures of inflammation,^4^, ^39^ macrophage infiltration did not decrease 3 months after VSG‐induced weight loss in the present study. These results are in line with the observation that macrophages accumulate in adipose tissue early during weight loss, likely because of an increase in adipose tissue lipolysis.^23^ Although inflammation may drive metabolic dysfunction in obesity, macrophages involved in controlled inflammation may play a distinct role in weight loss that allows for physiological adipose tissue remodelling.^52^

CLSs have been associated with obesity and insulin resistance. However, most of the studies that have investigated CLS have been performed in mice, and only a handful of studies have looked at CLS density after rapid weight loss in humans. In one study of patients who underwent RYGB‐induced weight loss, there was a reduction in CLS in patients with type 2 diabetes but not in euglycaemic patients.^53^ Interestingly, a study looking at postmenopausal women undergoing rapid weight loss by caloric restriction found an increase in CLS density in subcutaneous adipose tissue.^54^ This study found that there was no difference in CLS density from a mean of six patients before surgery compared with 3 months after VSG‐induced weight loss. The finding that increased CLS density occurs in the setting of obesity supports that CLS formation may represent increased cell death from increased adipocyte hypertrophy.^24^ In the setting of weight loss, it may represent increased macrophage accumulation because of increased lipolysis.^23^ Alternatively, the development of CLS in obese adipose tissues may signify that once they are formed, they persist in adipose tissue even after weight loss. Taken together, these data suggest CLS accumulation is a dynamic process in humans. Early weight loss after VSG is associated with a reduction in adipocyte size and a decline in IL‐6 gene expression levels at the local adipose tissue level but an increase of IL‐6 at the systemic level. The findings presented here suggest that while IL‐6 produced by adipose tissue is associated with obesity, systemic levels of IL‐6 may be associated with weight loss and improved insulin sensitivity early after VSG‐induced weight loss. Macrophage infiltration and CLS density are similar in adipose tissue derived from patients with obesity after VSG‐induced weight loss, indicating that ATMs may have a different function in the setting of weight loss.

The major limitation of this study was the small sample size; however, these findings add to the growing body of literature demonstrating that adipocyte cell hypertrophy is associated with obesity and that adipocyte hypertrophy decreases after VSG‐induced weight loss.^55^ Importantly, this study was conducted in mostly African American women, a currently understudied population. The contribution of inflammation or other factors may differ between patients because of various factors, including genetics. Given that obesity and type 2 diabetes affect non‐Hispanic black women with higher prevalence,^56^ inclusion of these patients in prospective studies is paramount to increase the understanding of mechanisms involved in metabolic dysregulation.

## CONFLICT OF INTEREST

The authors declare that the research was conducted in the absence of any commercial or financial relationships that could be construed as a potential conflict of interest.

## AUTHOR CONTRIBUTIONS

I.C. and M.J.B. conceived the experiments and analysed the data. All authors listed made substantial, direct, and intellectual contribution to the work and approved it for publication.

## FUNDING INFORMATION

T32DK007011 to I.C., R01 DK103014 to M.J.B., and P30 DK020595 from the University of Chicago, Diabetes Research and Training Center (DRTC).
